# Supplementary material for: Wavelength Dependence of the Transformation Mechanism of Sulfonamides Using Different LED Light Sources and TiO2 and ZnO Photocatalysts
Source: Materials (Basel). 2021 Dec 22;15(1):49. doi: 10.3390/ma15010049 (PMC8745830; doi:10.3390/ma15010049)
Supplement: Supplementary file 1 [file materials-15-00049-s001.zip › materials-1517565-supplementary.pdf]

Supplementary materials

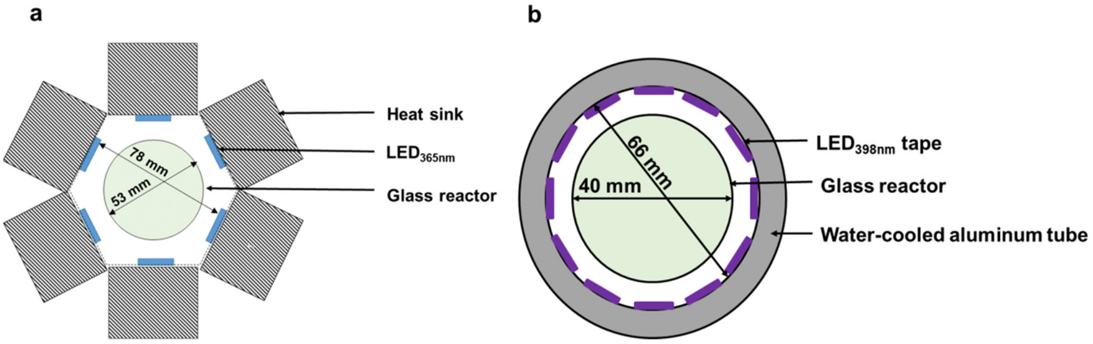

Figure S1. The schematic figure of the photoreactors ((**a**): LED_{365nm}, (**b**): LED_{398nm})

Table S1. The list of used chemicals, their distributors and purity

Chemical	Distributor	Purity
COU	Sigma Aldrich (St.Louis, Missouri, USA)	99%
1,4-BQ	Acros Organics (Geel, Belgium)	99%
SMT	Sigma Aldrich (St.Louis, Missouri, USA)	99%
SMP	Fluka (Buchs, Switzerland)	99%
NaCl	VWR (Radnor, Pennsylvania, USA)	99%
$NaHCO_3$	VWR (Radnor, USA)	99%
HCl	Sigma Aldrich (St.Louis, Missouri, USA)	98%
NaOH	VWR (Radnor, Pennsylvania, USA)	99%
Catalase (bovine liver)	Sigma Aldrich (St.Louis, Missouri, USA)	>55% protein content
$Fe_2(SO_4)_3 \times nH_2O$	VWR (Radnor, Pennsylvania, USA)	98%
$K_2C_2O_4$	Reanal (Budapest, Hungary)	98%
1,4-phenantroline	Sigma Aldrich (St.Louis, Missouri, USA)	99%
N_2	Messer Hungary (Budapest, Hungary)	99.995%
Synthetic air	Messer Hungary (Budapest, Hungary)	Medical grade
Formic acid	VWR (Radnor, Pennsylvania, USA)	100%
MeOH	VWR (Radnor, Pennsylvania, USA)	99.8%
H_2O	Merck-Millipore (Burlington, Vermont, USA)	ultrapure

Table S2. The measured, relevant parameters of the real water matrices

Parameter	Tap water	Biologically treated domestic wastewater
pH	7.4	7.8
Conductivity ($\mu S\ cm^{-1}$)	627	1258
COD ($mg\ dm^{-3}$)	4.2	24.4
NH_4^+–N ($mg\ dm^{-3}$)	<0.4	<0.4
NO_3^- ($mg\ dm^{-3}$)	<0.7	3.37
Cl^- ($mg\ dm^{-3}$)	8.75	120
TOC [1] ($mg\ dm^{-3}$)	0.79	6.9
TIC [2] ($mg\ dm^{-3}$)	73.4	103.4

[1] Total Organic Carbon
[2] Total Inorganic Carbon, the sum of dissolved CO_2, HCO_3^- and CO_3^{2-}

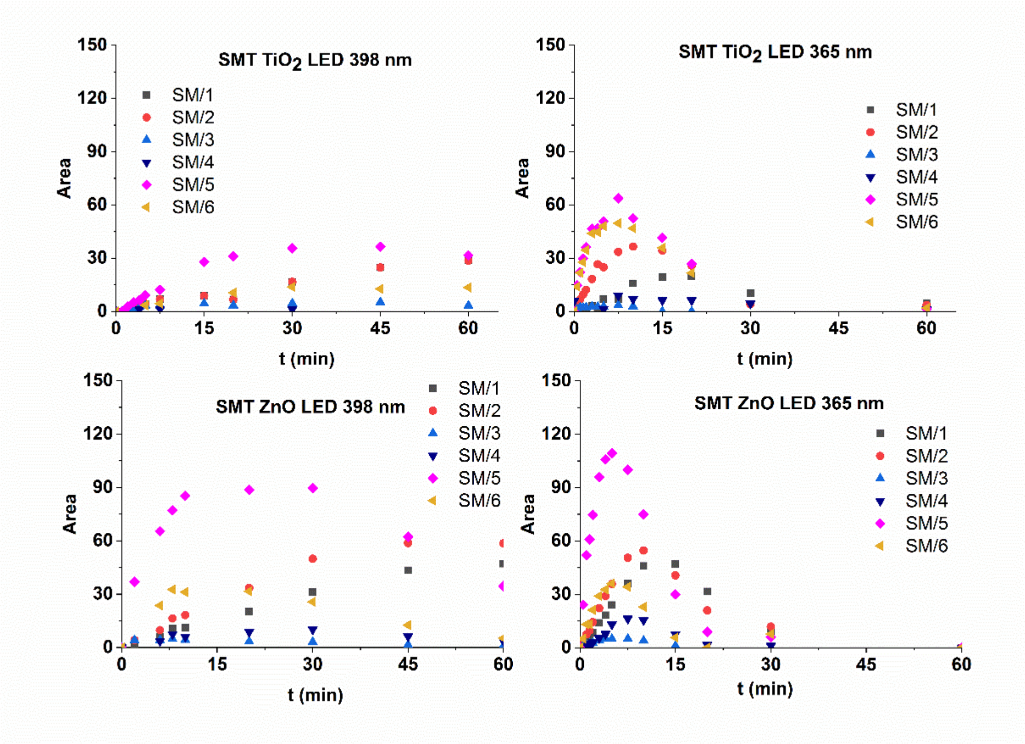

Figure S2. The peak area (HPLC-DAD) of the products of SMT transformation as a function of irradiation time

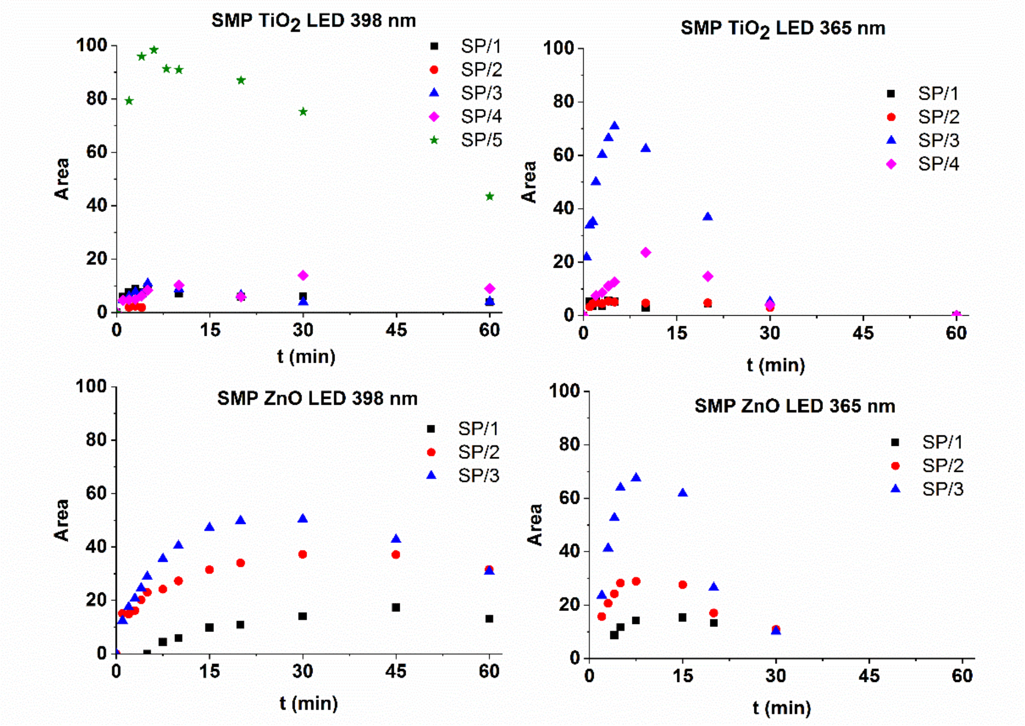

Figure S3. The peak area (HPLC-DAD) of the products of SMP transformation as a function of irradiation time

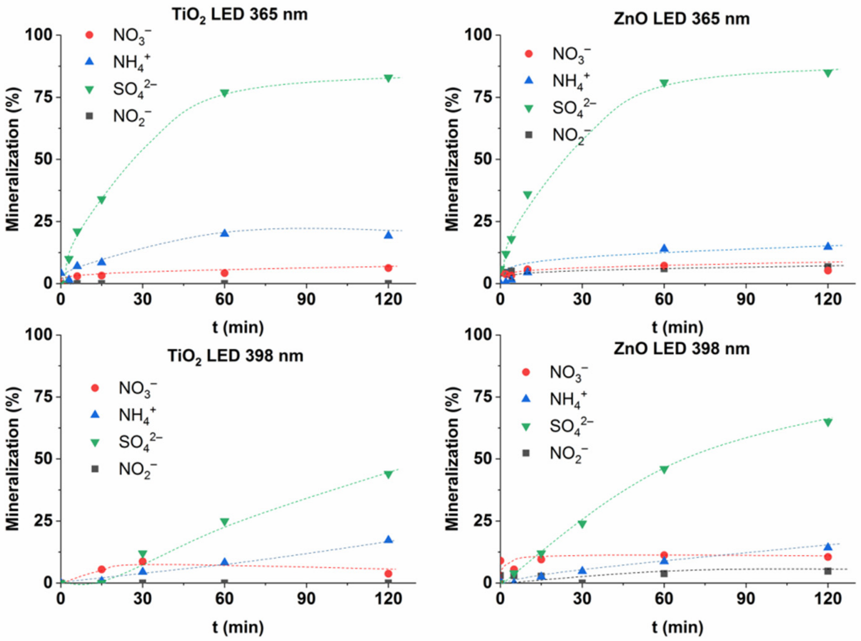

Figure S4. The transformation of organic nitrogen and sulfur to inorganic ions during the photocatalytic removal of SMT (Mineralization(%) = concentration of given ion divided by the organic N-content or S-content of the starting solution)

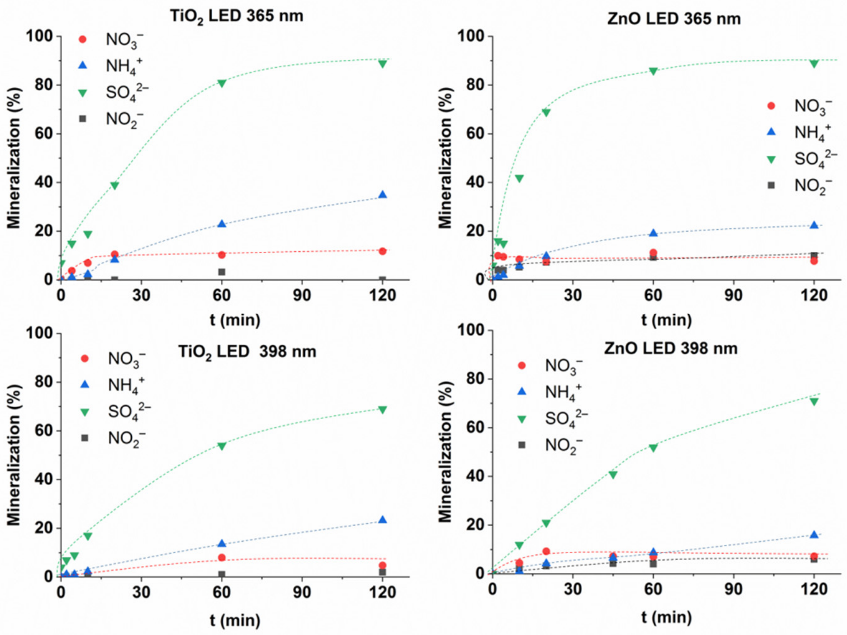

Figure S5. The transformation of organic nitrogen and sulfur to inorganic ions during the photocatalytic removal of SMP (Mineralization(%) = concentration of given ion divided by the organic N-content or S-content of the starting solution)
